# AI-Empowered Electrochemical Sensors for Biomedical Applications: Technological Advances and Future Challenges

**DOI:** 10.3390/bios15080487

**Published:** 2025-07-28

**Authors:** Yafeng Liu, Xiaohui Liu, Xuemei Wang, Hui Jiang

**Affiliations:** State Key Laboratory of Digital Medical Engineering, School of Biological Science and Medical Engineering, Southeast University, Nanjing 210096, China; lyafeng@seu.edu.cn (Y.L.); 101013182@seu.edu.cn (X.L.)

**Keywords:** artificial intelligence (AI), electrochemical biosensors, disease diagnosis, drug development, precision medicine

## Abstract

Biomarkers play a pivotal role in disease diagnosis, therapeutic efficacy evaluation, prognostic assessment, and drug screening. However, the trace concentrations of these markers in complex physiological environments pose significant challenges to efficient detection. It is necessary to avoid interference from non-specific signals, which may lead to misjudgment of other substances as biomarkers and affect the accuracy of detection results. With the rapid advancements in electrochemical technologies and artificial intelligence (AI) algorithms, intelligent electrochemical biosensors have emerged as a promising approach for biomedical detection, offering speed, specificity, high sensitivity, and accuracy. This review focuses on elaborating the latest applications of AI-empowered electrochemical biosensors in the biomedical field, including disease diagnosis, treatment monitoring, drug development, and wearable devices. AI algorithms can further improve the accuracy, sensitivity, and repeatability of electrochemical sensors through the screening and performance prediction of sensor materials, as well as the feature extraction and noise reduction suppression of sensing signals. Even in complex physiological microenvironments, they can effectively address common issues such as electrode fouling, poor signal-to-noise ratio, chemical interference, and matrix effects. This work may provide novel insights for the development of next-generation intelligent biosensors for precision medicine.

## 1. Introduction

With the escalating focus on health in modern society, the demands for early disease diagnosis, treatment, and prevention have experienced a notable surge. Critical illnesses such as AIDS [1], coronavirus disease 2019 (COVID-19) [2], and cancer [3] pose severe threats to public health security and human well-being. Furthermore, the global aging population has exacerbated the need for continuous monitoring of chronic diseases, including cardiovascular disorders and diabetes, among the elderly [4,5,6]. Achieving early disease diagnosis remains a persistent objective and significant challenge for both clinical and basic medicine. In the realm of biological detection, biomarkers, as indicators of human biological processes, are extensively involved in various physiological activities. Among these, disease markers constitute a crucial category of biomarkers, being intimately associated with the onset and progression of diseases [7,8,9]. Currently, disease markers are increasingly utilized in patient management, encompassing disease diagnosis and evaluation of therapeutic efficacy and prognosis, and they also play pivotal roles in pathogenic mechanism research and drug screening. Biomarkers can be classified according to multiple dimensions, including molecular type, function, source, and disease type. For example, the expression of circulating tumor DNA, as a genomic biomarker, can be used for cancer detection [10]. Glial fibrillary acidic protein in serum is a potential stroke biomarker, and changes in its concentration can be used for monitoring stroke patients [11]. In addition, some small-molecule metabolites (such as glucose, low-density lipoprotein cholesterol, and creatinine) can be used for the diagnosis of chronic diseases such as diabetes, cardiovascular diseases, and kidney diseases [12,13,14]. However, the concentrations of biomolecules that often need to be detected are extremely low, rendering it difficult to accurately identify them with traditional detection methods [15]. Given the significant physiological significance of disease markers, the realization of their precise, sensitive, and specific detection holds paramount importance for the development of the biomedical field.

A biosensor is a detection device that combines biological recognition elements with signal conversion technology. It can measure specific chemical or biological molecules with high sensitivity and selectivity. It uses biological components (such as artificial mimic enzymes) to recognize target substances. Then, it converts tiny changes generated during the recognition process into measurable signals (such as electrical signals, optical signals, etc.) through physical or chemical methods. Finally, it outputs the detection results [16,17]. Common detection methods for gene expression levels, protein levels, and small-molecule metabolites (such as glucose, urea, and creatinine) include colorimetry [18], fluorescence [19], surface-enhanced Raman spectroscopy (SERS) [20], and surface plasmon resonance (SPR) [21]. There are also methods like mass spectrometry and microfluidic chips to achieve the detection of markers [22,23]. Compared with other detection methods, electrochemical biosensors have been widely used for substance concentration measurement and show great application potential in disease marker detection due to their characteristics of fast response [24], high sensitivity [25], low cost [26], and ease of quantification [27]. In electrochemical biosensors, signals are typically triggered by electron or ion transfer on conductive transducers through biological recognition processes [28]. The molecular recognition element, as the core component of electrochemical biosensors, exhibits sensitivity and selectivity towards the targets, enabling specific recognition and interaction with the targets, which in turn causes changes in the state of the electrode surface [29]. The electrode, as a conversion element, outputs the perceived changes in the form of electrochemical signals (current, potential, impedance, charge), and the signals are amplified through transducers to achieve quantitative detection of the targets [30,31]. Currently, to achieve personalized medical and health management, researchers have developed a series of electrochemical biosensors. These sensors aim to realize real-time, rapid, and convenient detection of disease biomarkers such as protein levels and small-molecule metabolites. This expands clinical testing from professional laboratories to public places. At present, the biosensors approved by the U.S. Food and Drug Administration (FDA) are mainly limited to two categories: blood glucose monitors and cardiovascular monitors [32]. Although a variety of electrochemical sensors can accurately, sensitively, and specifically detect disease-related metabolite molecules in a range of biological fluids (such as serum [33], urine [34], sweat [35], saliva [36], and cerebrospinal fluid [37]), the development of electrochemical biosensors in biomedical applications still faces challenges, especially in ensuring the stability of electrode modification materials, the accuracy of electrical signal conversion, and the reproducibility of results.

The vigorous development of artificial intelligence (AI) has provided new opportunities for the field of biosensing science. Machine learning (ML), as the core of AI [38], addresses the computational problem of automatic improvement through experience. The latest advancements in ML are driven by the continuous explosion of new learning algorithms and theoretical developments, as well as the availability of online data and low-cost computing. ML relies on different algorithms to solve data problems, and common ML algorithms include supervised learning [39], decision trees [40], support vector machines (SVM) [41], and unsupervised learning [42]. AI technology based on ML is a powerful tool for complex data analysis and information mining, which provides enormous potential opportunities for material screening [43], synthesis [44] and characterization [45], performance prediction [46], parameter optimization [47], and device assembly [48], and has rapidly become a new favorite in assisting biosensing science research. For example, Xu et al. developed a nanozyme and bioenzyme dual-conjugated fluorescence sensor array for the detection of amyloid proteins [49]. They used an ML algorithm for feature value selection to select and optimize sensor elements, and combined with a dimensionality reduction ML algorithm to achieve the differentiation of different amyloid peptides. Yu et al. developed a fluorescence sensor array composed of negatively charged poly(p-aryl acetylene) (PPE) and positively charged aggregation-induced emission (AIE) fluorophores for multiplex bacterial identification [50]. Combined with ML algorithm optimization, it is worth noting that the random forest (RF) model achieved 96.7% accuracy in distinguishing clinical urinary tract infection samples. Materón et al. designed a plasmonic biosensor by functionalizing the surface of gold nanoparticles with polyclonal antibodies for the detection of SARS-CoV-2 [51]. Gold nanoparticles exhibit a high surface density of free electrons. The surface plasmon resonance, generated by the collective oscillation of surface electrons induced by visible light, manifests as an extinction band in the visible light region. When the antibodies conjugated to gold nanoparticles bind to viral antigens, clusters form on the virus surface, leading to the aggregation of nanoparticles. This causes a significant change in their color. The collected images of color changes are processed and analyzed using a machine learning algorithm based on finite-difference time domain (FDTD). This enables 100% accurate diagnosis of COVID-19 using only 20 μL saliva samples. Dizaji et al. proposed a gold nanorod array as a surface-enhanced infrared absorption spectroscopy platform for bacterial detection [52]. The obtained spectral signals, combined with ML algorithms such as principal component analysis (PCA), hierarchical cluster analysis (HCA), linear discriminant analysis (LDA), and SVM classification, realize label-free identification of *E. coli*, *Staphylococcus aureus*, and *Bacillus subtilis*.

Although traditional ML techniques have been widely applied in the field of biosensing and demonstrate prominent advantages in computational and development speed, their evaluation performance for complex biological samples—particularly those with highly structured data features—leaves much to be desired. This is mainly because traditional machine learning algorithms rely on manual data extraction. Artificially designed features struggle to fully capture complex patterns [53,54]. For example, the concentrations of metabolic biomarkers (such as lactic acid and glucose) change dynamically with the microenvironment. Traditional static models cannot capture real-time fluctuations and are susceptible to interference from sample batch effects [55,56,57]. With the continuous development of AI technologies, traditional ML is gradually evolving toward deep learning (DL) and artificial neural networks (ANNs) [58,59]. In recent years, deep neural networks (DNNs), as a popular branch of ML, have emerged. DNN represents a model capable of autonomously extracting features from input data, conducting self-learning, algorithm selection, and optimized training [60,61]. By controlling the size and complexity of the model, it is possible to achieve predictive accuracy at any desired level. ANNs, as mathematical algorithms that transform inputs into outputs in a specific manner, constitute the basic structural units of neural networks (NNs). Each neuron can be connected in different ways according to the model design. Notably, input neurons signify the number of feature values calculated from the data input into the network, and each connection between a pair of neurons represents a trainable weight parameter [62]. Currently, several novel NN models have been designed for application in biological detection research, including multi-layer perceptron (MLP), convolutional neural networks (CNNs), recurrent neural networks (RNNs), image convolutional networks, and autoencoders [63,64,65,66,67]. Among these, CNN is a DL algorithm widely used in fields such as image, video, and natural language processing. In recent years, CNN has achieved remarkable success in analyzing and processing various large biological databases, demonstrating strong application potential in the field of biosensing. Wang et al. [68] reported a label-free SERS detection method based on aptamer-guided gold nanoparticle enhancement and a CNN classification algorithm, achieving rapid identification of *Methicillin-sensitive Staphylococcus aureus* and *Methicillin-resistant Staphylococcus aureus* with an accuracy as high as 100%. Cheng et al. developed a biosensor for serum-based liver disease detection, composed of zinc oxide nanorods decorated with gold–silver nanocomposites [69]. By integrating CNN to recognize serum SERS spectra, this sensor enables rapid detection of liver diseases with a predictive accuracy of 97.78%.

**Figure 1:**
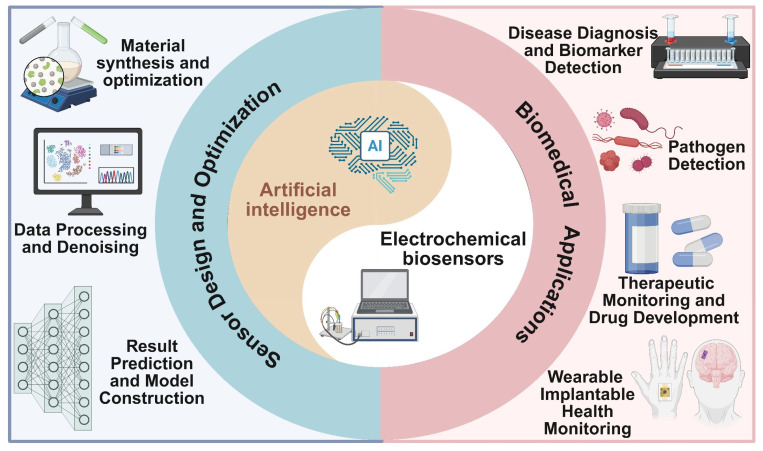
AI-assisted electrochemical biosensors for biomedical application (created in BioRender).

The current empowerment of AI serves as an emerging tool for the design, preparation, characterization, performance evaluation, and application research of sensor modification materials across diverse domains. Compared with traditional optimization experimental design schemes, ML enables the rapid realization of multivariate parameter synchronous optimization and material screening for novel sensor composite materials, as well as the exploration of the relationship between sensor performance and components. AI technology not only serves as a technical support to facilitate the rapid development of the electrochemical sensing field but also acts as a technical guarantee to achieve accurate and convenient monitoring of target substances. This paper reviews the development of AI technology in electrochemical biosensors for biomedical applications (Figure 1), along with the current challenges and potential application prospects, providing a new perspective for the future development of intelligent electrochemical biosensors.

## 2. Key AI Technologies in Electrochemical Sensors

### 2.1. Sensor Design and Material Optimization

The application of AI for biosensor design first manifests in material selection and optimization. The performance of biosensors is highly dependent on their core material systems, including electrode substrate materials, interface modification materials, and biosensitive membranes. The physicochemical properties of these materials (such as conductivity, specific surface area, and biocompatibility) directly affect the sensitivity, selectivity, stability, and response speed of the sensor. Among them, the electrode is the basis for signal conversion in biosensors. Its material selection directly influences electron transfer efficiency and background noise control. Modifying the electrode surface can increase the specific surface area, accelerate electron transfer, and enhance biomolecule immobilization. In addition, the biosensitive membrane provides specific recognition capabilities. Its design can determine the selectivity and anti-interference ability of the sensor [70,71,72]. AI can predict the impact of different material combinations on sensor performance by analyzing vast amounts of material data and performance parameters. For instance, ML models can forecast the potential properties of new materials and guide material selection and synthesis by learning from the electrochemical characteristics and structural information of known materials [73,74]. This approach not only accelerates the discovery of new materials but also optimizes the performance of existing materials, thereby enhancing the sensitivity, selectivity, and stability of biosensors.

Mainstream electrode materials are relatively single. Due to their high conductivity, stability, and mature manufacturing processes, current electrodes mainly rely on materials such as gold, platinum, and carbon-based materials (e.g., graphene, carbon nanotubes) [75]. However, although flexible materials (such as MXene/polymer composites) or nanoenzyme materials have excellent performance, it is difficult to widely apply them in sensor production due to complex mass production processes and high costs [70,76]. This leads to a limitation in the sensitive electrode materials of existing sensors, which means that only a small number of sample data can be used for the training of machine learning models.

**Figure 2:**
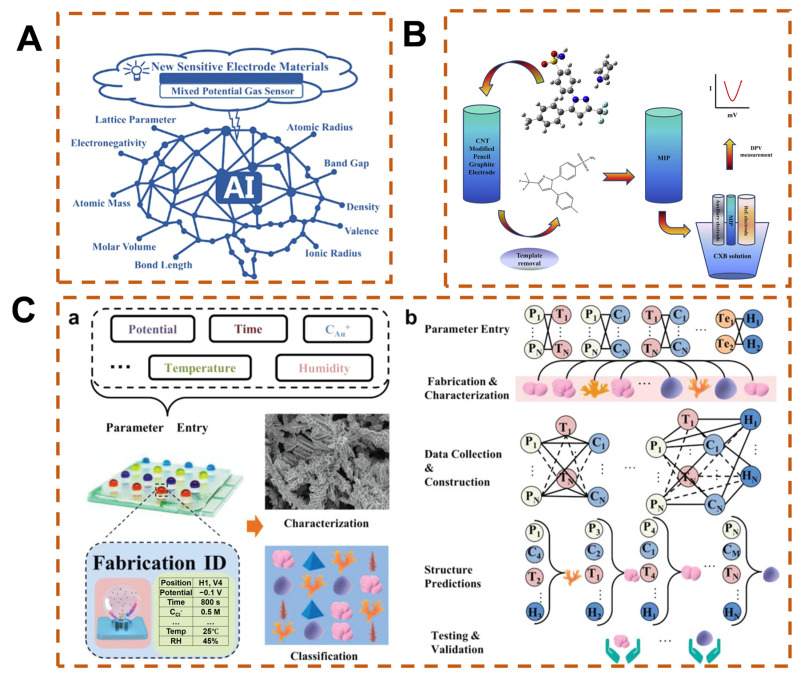
Development and optimization of AI-enhanced sensors. (**A**) Tree-based ML models for screening sensitive electrode materials [78], Copyright © 2021 American Chemical Society. (**B**) AI-optimized parameters for MIP sensors [81], Copyright © 2017 Elsevier B.V. (**C**) Predictive fabrication of gold nanostructures via microdroplet array platform and ANNs [89], (a) Application of multiparameter modulation in the preparation and characterization of gold nanostructures, (b) Integrated electrochemical deposition technology based on ANNs. Copyright © 2020 WILEY-VCH Verlag GmbH & Co. KGaA, Weinheim.

Tree-based ML models (e.g., decision trees, RF) exhibit unique advantages in feature selection [77]. Their core mechanism lies in using information entropy to measure feature importance, automatically evaluating the contribution of each feature to the prediction result through the splitting process of decision nodes, thereby achieving feature ranking and screening. These characteristics endow tree models with both prediction efficiency and model transparency in small-sample scenarios such as material property prediction, providing reliable theoretical support for material design. Wang et al. constructed a structured dataset of sensitive electrode materials for potentiometric sensors using tree-based ML models, using the sensitivity of materials to nitrogen dioxide as labels (Figure 2A) [78]. The sensitive electrode materials screened through model training demonstrated excellent sensing performance. To achieve optimal sensor performance, careful optimization of key parameters during the fabrication process is essential. For example, in the manufacturing of electrochemical biosensors based on molecularly imprinted polymers (MIPs), instrumental variables, synthesis solution conditions, and analyte dissolution conditions are the primary factors for optimization [79]. Yarahmadi et al. [80] utilized nonlinear regression algorithms (including classification and regression trees, support vector regression, and k-nearest neighbor (KNN)) and ensemble algorithms (such as gradient boosting and RF) to predict the quality of imprints. Another study reported the preparation of a MIP sensor on a functionalized carbon-nanotube-modified graphite electrode, combining central composite design, ANNs, and genetic algorithms for sensor design, modeling, and optimization (Figure 2B) [81].

Furthermore, nanomaterials have shown significant application values in sensing, optoelectronics, and biomedical fields, and particularly as modified materials for electrochemical sensors, attributed to their unique chemical and physical properties [82,83]. Their performances are highly contingent upon the regulation of multiple parameters (e.g., potential, temperature, ion concentration) during the preparation process, as minor parameter fluctuations can induce remarkable changes in morphology (size, shape, composition) [84,85]. Although traditional electrochemical deposition methods enable the fabrication of noble metal nanostructures through multi-parameter adjustment, they entail notable limitations: reliance on conventional strategies such as trial-and-error and random attempts, coupled with the requirement for large-volume solution systems, which culminate in prolonged research cycles, substantial reagent consumption, and low efficiency. ANNs, by constructing parameter optimization models, establish quantitative relationships between input parameters and output performances [86,87,88]. The integration of ANNs with high-throughput preparation technologies not only significantly shortens the material R&D cycle but also reduces reagent consumption, offering a novel paradigm for the efficient development of novel functional nanomaterials. Song et al. demonstrated a multi-parameter regulated electrodeposition platform that enables the controlled synthesis of gold nanostructures via a microdroplet array reactor, and this platform, in conjunction with ANNs, facilitates prediction of the structural properties of as-prepared materials (Figure 2C) [89].

### 2.2. Data Processing and Denoising

Biochemical sensor data acquisition relies on diversified technologies such as electrochemistry, fluorescence, mass spectrometry, and SERS, which are capable of capturing the subtle features of biological/chemical processes and generating information-rich datasets that present analytical challenges [90]. Raw sensing data often suffer from issues including noise interference, outliers, and missing values, and systematic deviations or inconsistencies are prone to occur across measurement batches. If these issues are not addressed, they will significantly affect the accuracy of subsequent analyses [91]. It has been reported that biosensors constructed using surface-functionalized nanomaterials and polymer coatings can reduce the interference by non-target biological components in complex body fluid matrices during continuous monitoring, thereby decreasing the sensor’s signal-to-noise ratio (SNR) and enhancing its accuracy and sensitivity [92,93]. Although hardware improvements based on advanced materials show potential in optimizing the performances of biosensors [94,95], especially electrochemical biosensors, their broad application in point-of-care testing (POCT) still faces some challenges. Compared with the precise temperature and humidity control and anti-interference conditions in laboratories, sensors in POCT scenarios are susceptible to environmental fluctuations, leading to the attenuation of biological molecular activity (such as an increase in enzyme inactivation rate) and the instability of electrochemical reaction kinetics, which in turn causes nonlinear signal drift [96]. AI algorithms such as ML, DL, and NN can improve data quality and integrity through pre-analysis data optimization, generating pure and concise datasets, effectively enhancing the expression of real biochemical signals and trends, and reducing the masking effect of human interference or noise [97,98].

In the signal processing of AI-assisted biosensors, the key is to remove abnormal eigenvalues and intelligently select only specific valid data to obtain stable descriptors. These descriptors can extract output information with analytical value during the identification, authentication, and quantification of markers. This avoids the problem of poor repeatability caused by factors such as electrode modification, electrode fouling, and matrix effects [95]. For example, Fatima et al. prepared an electrochemical electrode with silver nanoparticles impregnated in multi-walled carbon nanotubes for the monitoring of antiviral drugs (acyclovir) [99]. ML algorithms were trained on experimental datasets, including pH, drying time, the concentration of prepared materials deposited on the working electrode, and voltammetric current response. The results showed that the ML-optimized electrochemical sensor not only has advantages such as reliable linear response, high sensitivity, and low detection limit but also exhibits reliable repeatability in the monitoring of acyclovir in actual urine samples.

**Figure 3:**
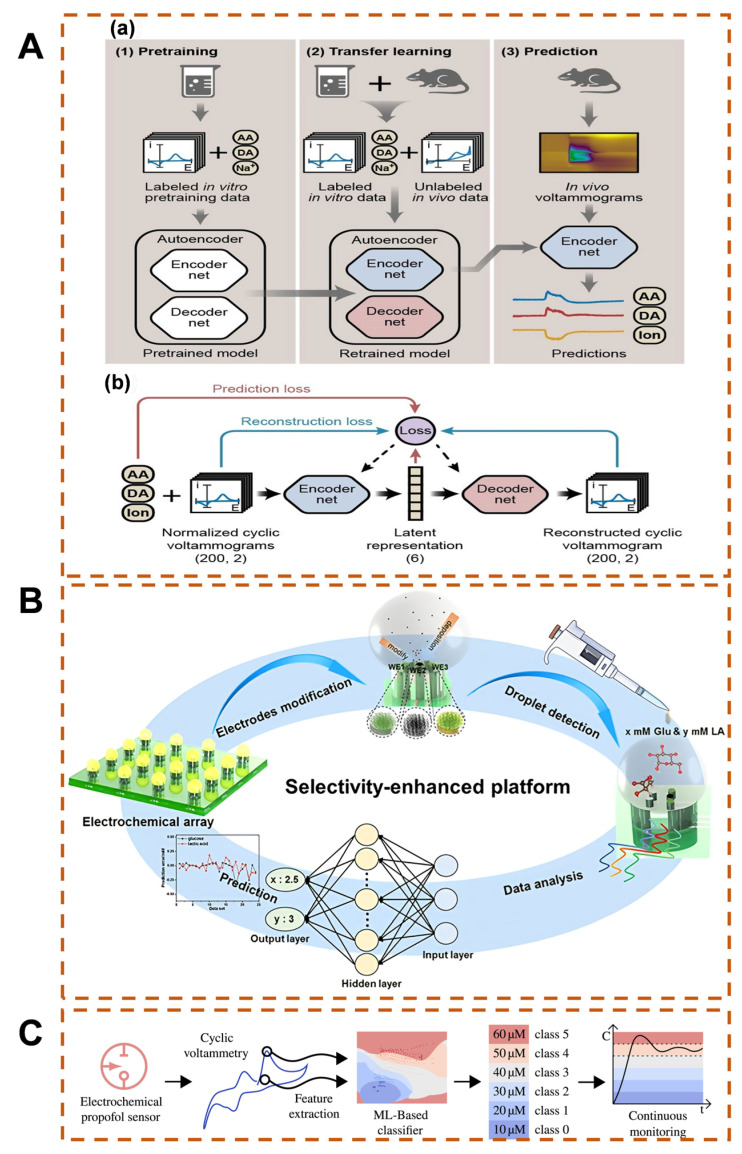
(**A**) DL-assisted voltammetric sensing platform for in vivo multiplexed neurochemical detection in the brain [100], (a) Workflow of DL for voltammetric sensing, (b) The autoencoder training progress. Copyright © 2021 John Wiley & Sons, Inc. or related companies. (**B**) NN models enhancing selectivity of non-enzymatic electrochemical biosensors for mixture analysis [103], Copyright © 2022 American Chemical Society. (**C**) ML-compensated monitoring of propofol amid electrode fouling effects [97], Copyright © 2020 Elsevier B.V.

DL algorithms, by constructing multi-layer nonlinear feature extraction networks, can break through the limitations of traditional feature engineering, excavate high-dimensional correlation features from complex functions, and significantly improve the analytical ability of complex samples, providing an effective detection means for multiplexed detection of clinical samples. Xue et al. constructed a DL voltammetry sensing platform to achieve high spatiotemporal resolution simultaneous monitoring of multiple neurochemicals (dopamine, ascorbic acid, and key electrolyte ions) in the living brain (Figure 3A) [100]. In terms of algorithm architecture, a self-encoder-like DNN was designed, integrating an encoder with three-level convolutional layers and a collaborative decoder network; a semi-supervised learning framework was introduced by using labeled in vitro data to pre-train the model and combining unlabeled in vivo data for transfer learning, which significantly improves feature generalization ability. NN models have been proven to handle problems such as complex structure and performance mapping through multi-level nonlinear transformations [89,101]. However, they rely on massive data to achieve high-precision perception and prediction, but the high cost and time-consuming nature of data collection have become the core bottleneck for their widespread application [102]. In electrochemical biosensors, the combination of the nonlinear modeling ability of NNs and high-throughput electrochemical microarrays can achieve low-cost batch data collection through parallel detection. Zhou et al. constructed a backpropagation neural network (BPNN)-driven non-enzymatic electrochemical sensing platform for the detection of glucose and lactate (Figure 3B) [103]. Among them, three modified electrodes with differentiated electrochemical properties were prepared and integrated into an electrochemical microdroplet array, which can simultaneously collect multi-channel currents to achieve low-cost collection of rich data. Based on the different response characteristics of three non-enzymatic working electrodes, the team used a BPNN to calculate the correct mapping relationship between current intensity and analyte concentration. The obtained 120 sets of current fluctuation data were used as the input layer of the network. BPNN simulates three basic functions of biological neurons: weighting, summation, and transmission. The output of the previous layer is the input of the next layer. This further identifies and predicts the concentrations of glucose and lactic acid in multi-analyte mixtures. Ultimately, it solves the selectivity bottleneck of non-enzymatic electrochemistry caused by overlapping oxidation peaks of glucose and lactic acid. The phenomenon of electrode surface fouling by biomacromolecules or suspended particles in the environment or measurement medium greatly limits the in vivo and in vitro analytical performance of sensors [104]. For example, during the continuous electrochemical detection of propofol, a commonly used clinical anesthetic, the applied potential difference causes propofol to lose electrons to generate free radicals, which further react with oxygen to form a polymer film attached to the electrode surface, ultimately restricting the sensor’s stability and accuracy [105]. To overcome this problem, Aiassa et al. used an ML algorithm based on a Gaussian radial basis function support vector classifier (RBF-SVC) to compensate for the pollution effect of propofol on the electrodes, to correct the sensor’s signal drift, and to achieve 100% classification accuracy in serum samples (Figure 3C) [97].

Overall, in addition to optimizing the hardware facilities of biosensing systems through the use of new materials or material modification to improve sensor performance, AI algorithms can also be employed for noise reduction and feature information extraction of electrochemical sensing signals, as well as automatic calibration of electrode signal deviations [95,106]. AI algorithms can mitigate the impacts of electrode fouling, variability, noise, and matrix effects in electrochemical biosensing measurements, thereby contributing to the enhancement of sensor sensitivity and specificity [107].

### 2.3. Result Prediction and Model Construction

**Figure 4:**
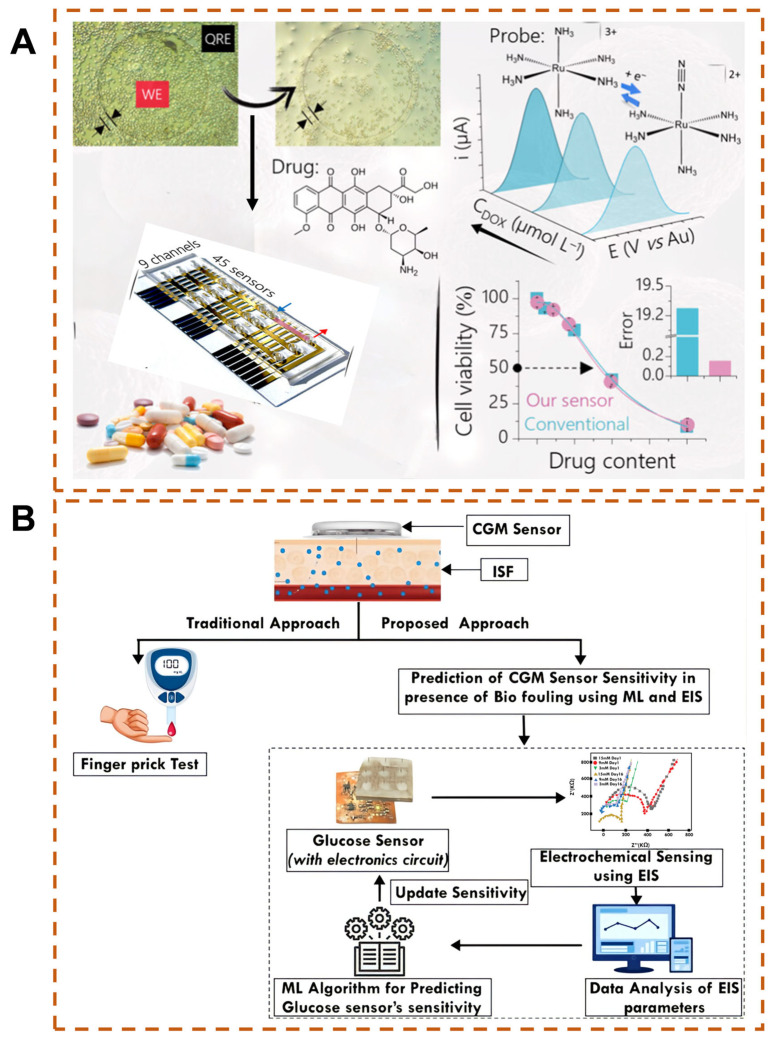
(**A**) SISSO-driven prediction of cell viability via high-throughput electrochemical systems [112], Copyright © 2024 American Chemical Society. (**B**) ML and electrochemical impedance spectroscopy (EIS) for predicting glucose sensor sensitivity and biofouling [113], © Copyright 2025 IEEE.

The optimized clean data can provide reliable inputs for subsequent AI analyses such as visualization, modeling, and classification, enhancing the validity and reliability of analysis results and reducing the impact of erroneous data on conclusions. In electrochemical biosensors, AI algorithms can also combine electrochemical data with other data modalities to improve prediction performance and directly provide actionable outputs for users [108]. Sure Independence Screening and Sparsifying Operator (SISSO) is an ML method that integrates symbolic regression and sparse optimization. It screens key physicochemical descriptors (such as electrochemical active area, electron transfer coefficient, and interface capacitance) from high-dimensional features. Through symbolic regression, it generates interpretable mathematical expressions (such as linear or nonlinear equations), avoiding the problem of non-interpretability of traditional black-box models (such as NNs). SISSO reduces the dependence on data volume through physics-guided feature construction. Moreover, the sparsity constraint of SISSO can filter the noise signals at the electrode interface, separate the characteristic peaks of target molecules from complex electrochemical signals, and improve the signal-to-noise ratio [109,110,111]. When Doretto and colleagues used a high-throughput electrochemical system to screen anticancer drugs (Figure 4A), in order to improve the accuracy of cell viability determination, they adopted the supervised ML method of SISSO, using the viability of cells at different drug concentrations as model output values and the current values obtained by square wave voltammetry as input variables. The prediction result for cell viability was 0.994 by the model, and its accuracy was significantly higher than that of conventional biochemical detection [112].

In addition, multimodal AI algorithms have been applied to continuously monitor electrochemical biosensors, improving performance by integrating operational status data with electrochemical data [114]. The research team used three ML techniques of linear regression, RF regressor, and decision tree, combined with electrical impedance parameters, to predict the sensitivity changes of biosensors (Figure 4B) [113]. Among them, linear regression is a supervised learning algorithm. In regression analysis, independent variables (explanatory variables) are the core input of the model. They are used to quantify the correlation with dependent variables (target variables) and generate prediction results based on this. Independent variables undergo significance testing and directionality judgment. This quantifies the direction and intensity of influence. By fitting data to construct a prediction equation, the regression model converts the combination of independent variables into predicted values [115]. The RF regressor is an ensemble technique that uses a large number of decision trees as well as bootstrapping and aggregation techniques to perform regression and classification tasks. The decision tree regression method helps divide the data set into more manageable subsets, which in turn facilitates the prediction process and provides more accurate results for the nonlinear distribution of the data set. The multimodal AI technology-assisted system accurately predicts blood glucose levels and adjusts according to the results after electrode fouling under time changes, thereby improving overall performance, compliance, and reliability.

## 3. Key Biomedical Applications of AI-Enabled Electrochemical Sensors

### 3.1. Disease Diagnosis and Biomarker Detection

In vitro diagnostics (IVD) refers to the qualitative and quantitative detection in human samples such as blood, body fluids, and tissues to determine diseases or physiological functions of the body. IVD has evolved into an indispensable medical tool for early disease diagnosis, prevention, continuous monitoring, and the treatment process. Electrochemical sensors, by virtue of their inherent advantages, possess enormous potential in the field of in vitro diagnostics. However, in vitro diagnostics rely on disease-related biomarkers, and both trace characteristics and diversity of disease markers significantly add difficulty to measurements [116]. The introduction of AI technology can not only improve the efficiency of data processing but also enhance the sensitivity and accuracy of sensors in the presence of biomatrices.

**Figure 5:**
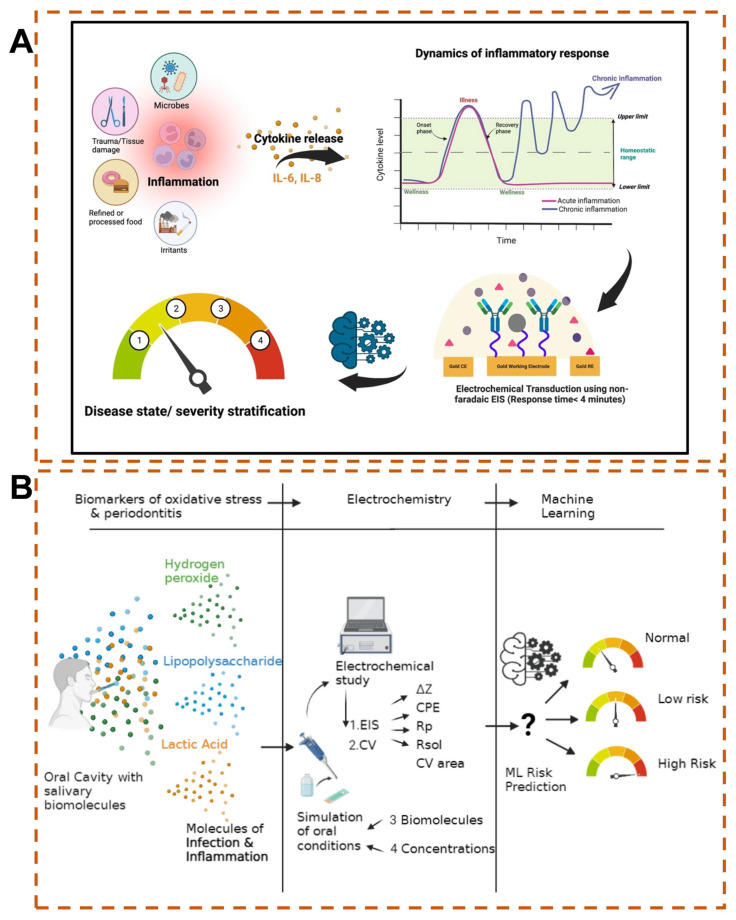
(**A**) RF algorithm integrated with electrochemical sensors for detection of inflammatory factors in urine samples [118], Copyright © 1996–2025 MDPI (Basel, Switzerland). (**B**) Intelligent salivary electrochemical biosensing for periodontitis detection [119], Copyright © 2025 Springer Nature.

Urine has been widely studied for quantifying inflammation, primarily attributed to its noninvasive acquisition, the stability of disease factors in urine samples, and the simplicity of sample processing, thus facilitating its use as a routine disease screening tool. The design of traditional electrochemical biosensors focuses on quantifying the concentration of biomarkers in urine. They achieve high-precision detection through electrochemical signal conversion but do not integrate disease grading algorithms. Moreover, urine composition is affected by factors such as water intake, drugs, and exercise, and the sensors have no built-in dynamic calibration modules [117]. Therefore, it is difficult for them to provide information or interpretations regarding the severity of diseases or the urgency of medical treatment. AI-empowered electrochemical biosensors can effectively overcome such issues. Ganguly et al. constructed an electrochemical sensor using standard planar gold three-electrodes, where monoclonal antibodies were employed to capture inflammatory factors (IL-6 and IL-8) for the detection of urine samples (Figure 5A) [118]. The output electrochemical impedance spectroscopy EIS was used for disease state classification. Combined with an ML model based on the RF algorithm, this approach achieved a prediction accuracy of 98% for disease state classification (Table 1).

Similarly, saliva is a safe, noninvasive, easily accessible biofluid without the risk of blood-borne disease transmission, pain, or discomfort, making it widely acceptable [126]. Saliva samples can be used to detect oral and salivary gland diseases. Periodontitis, a common oral disease, has a high global prevalence. Studies have confirmed that hydrogen peroxide, lipopolysaccharides, and lactic acid in saliva are important biomarkers of periodontitis [127,128,129]. George et al. developed an intelligent electrochemical biosensor based on ML for the diagnosis of periodontitis (Figure 5B) [119]. The sensor consists of a gold working electrode, a platinum counter electrode, and a silver reference electrode. EIS and cyclic voltammetry were used to measure hydrogen peroxide, lipopolysaccharides, and lactic acid in saliva. The obtained electrochemical parameters were combined with ML models including SVM, SVM with radial basis function (RBF) kernel, and NNs for risk prediction. The results demonstrated the high efficiency of the developed intelligent sensor in risk prediction.

**Figure 6:**
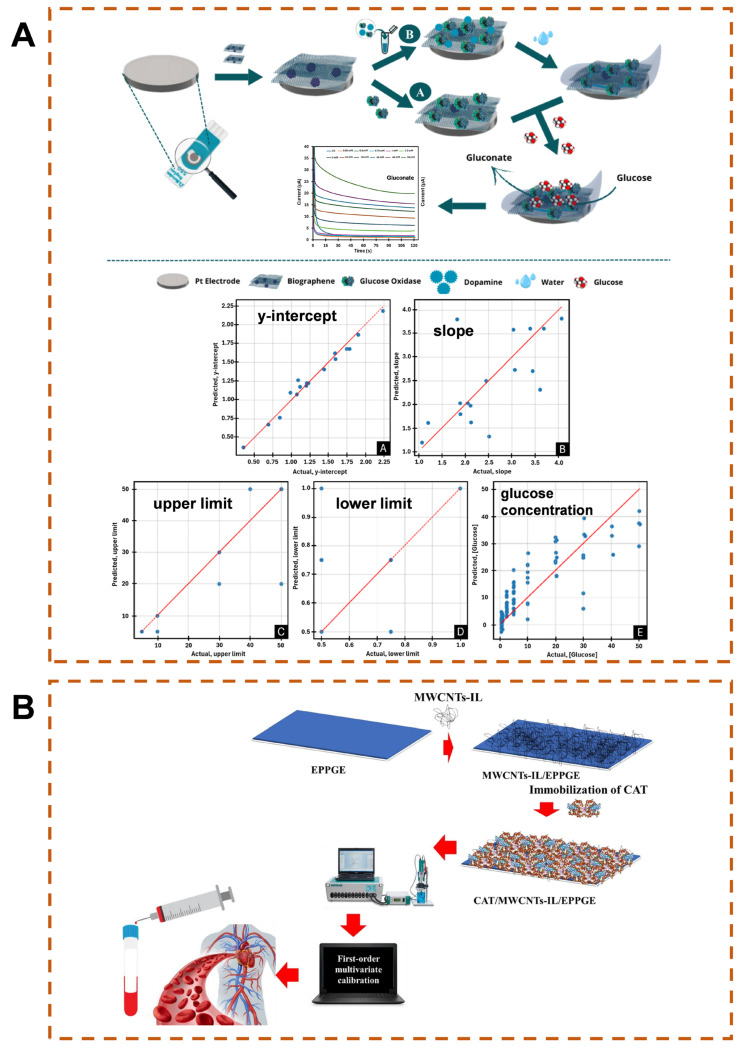
(**A**) ML-enhanced electrochemical biosensor for quantifying glucose in serum [120]. (**B**) Intelligent chemometrics-integrated electrochemical enzymatic biosensing platform for erythrocyte enumeration [121], Copyright © 2023 Published by Elsevier B.V.

Although blood sample acquisition is invasive, blood contains various nutrients, cellular metabolites, enzymes, antibodies, hormones, inorganic salts, etc., which can reflect the characteristics of the body’s internal circulation and individual physiological functions. With numerous detectable indicators, it is the most widely used in clinical practice. For example, Abreu’s team developed an intelligent electrochemical biosensor for detecting glucose concentration in serum samples (Figure 6A) [120]. The sensor uses graphene-modified platinum electrode, and then encapsulates the glucose oxidase layer through dopamine electropolymerization to enhance stability and electron transfer efficiency. The sensor has a wide linear detection range (0.75–40 mM), a low LOD of 0.078 mM, and a correlation coefficient of 0.988, making it suitable for complex serum environments. Moreover, under the optimization of ML algorithms, the decision tree algorithm accurately predicts calibration parameters (determination coefficient > 0.9), and the multi-layer perceptron (MLP) effectively correlates electrochemical signals with glucose concentration (determination coefficient 0.828), proving that ML can improve detection reliability (Table 1).

In addition, changes in blood cells can also reflect the health status of the body. Jalili and colleagues proposed an electrochemical biosensor for quantitative analysis of red blood cells (Figure 6B) [121]. The sensor uses a multi-walled carbon nanotube-ionic liquid modified pyrolytic graphite electrode and immobilizes catalase to amplify the oxygen reduction signal in red blood cells. Multivariate calibration models such as radial basis function–partial least squares (RBF-PLS), least squares–support vector machine (LS-SVM), and radial basis function–artificial neural network (RBF-ANN) are used to process voltammetric data to improve selectivity. RBF-ANN amperometry shows the best performance. This method provides a highly sensitive and selective solution for blood analysis (Table 1).

### 3.2. Infectious Diseases and Pathogen Detection

For a long time, infectious diseases have been emphasized as a major cause of damage to global public health [130]. Common bacterial infections include pneumonia, wound infections, urinary tract infections, etc. Although it must be acknowledged that non-bacterial factors (such as fungal infections, malaria, and HIV) also contribute to the global infection burden, reducing bacterial infectious diseases and their impact on health is a priority in addressing public health issues. The global bacterial infection burden report shows that in 2019, there were approximately 13.7 million deaths related to bacterial infections worldwide, accounting for 13.6% (10.1–18.1%) of global deaths, making it the second leading cause of death globally, after ischemic heart disease [131]. Accurate and timely detection of pathogens such as pathogenic bacteria and viruses is a crucial step in effectively controlling infectious disease activities. Traditional pathogen detection methods include culture, microscopic observation, biochemical tests, etc. These methods are usually time-consuming and complex to operate. For example, the culture method takes hours to days to obtain results, which may lead to delayed treatment in emergency situations. In contrast, biosensors can quickly and sensitively detect pathogens such as bacteria, viruses, and parasites, providing immediate results [132].

Bacterial detection is often constrained by on-site environmental complexities and the intrinsic heterogeneity of samples. In recent years, emerging research has demonstrated that electrochemical biosensing integrated with AI enables on-site detection of multiple pathogenic bacteria, featuring rapidity, effectiveness, sensitivity, and cost-efficiency [133,134]. Wang et al. proposed an ML-driven cell-imprinted electrochemical impedance sensor for qualitative and quantitative detection of three pathogenic bacteria [134]. The sensor constructs a bacterial-specific imprinted layer via electrochemical polymerization of 3-aminophenylboronic acid, integrating EIS parameters to establish datasets. An optimized RF classifier enables qualitative identification of bacterial species and semi-quantitative concentration analysis, achieving precise quantification within the range of 10^1^–10^6^ CFU/mL through normalized weighting.

**Figure 7:**
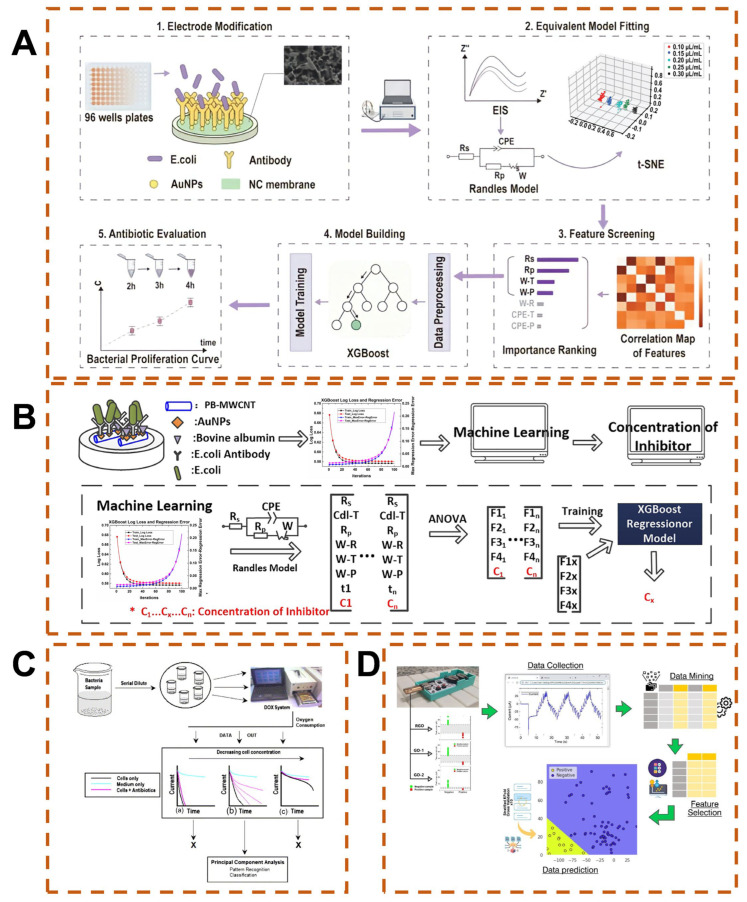
(**A**) ML-integrated electrochemical impedance profiling for bacterial proliferation-based antibiotic concentration evaluation [136], © Copyright 2025 IEEE. (**B**) XGBoost–EIS integrated sensor for determination of sub-inhibitory concentration effects on *E. coli* [122], Copyright © 2020 Elsevier B.V. (**C**) Multi-array intelligent electrochemical sensor with pattern recognition capability for bacterial detection and identification [137], (a–c) represent DOX responses for high medium and low cell concentrations, Copyright © 2006 Elsevier B.V. (**D**) ML-optimized electrochemical biosensor to enhance rabies virus detection performance [138], Copyright © 2024 Elsevier B.V.

Antimicrobial agents serve as a primary treatment for bacterial infections, yet the escalating antimicrobial resistance (AMR) poses a severe challenge to modern healthcare systems [135]. Controlling drug concentration and dosage is pivotal for microbial resistance research, where quantification of antibiotics and bacteriostatic agents can be indirectly achieved by measuring bacterial concentrations. Addressing the need for antibiotic efficacy assessment, Chen et al. developed an indirect electrochemical impedance-based detection method [136]. Through real-time monitoring of impedance variations in *E. coli* proliferation on AuNP-modified nitrocellulose membranes under the action of amikacin sulfate, combined with equivalent circuit model analysis for signal mechanism interpretation and t-SNE feature screening, an ML model was implemented to achieve precise prediction of antibiotic concentrations (Figure 7A). This method exhibited a prediction error of only ±2.9 × 10^−3^ μL/mL within 2–4 h after administration, providing a highly sensitive quantitative strategy for long-term tracking of low-concentration antibiotic efficacy. Another study proposed an intelligent assisted EIS biosensor strategy for quantitative evaluation of the inhibitory effect of hydrogen peroxide on *E. coli* growth (Figure 7B) [122]. By capturing bacteria on antibody-modified electrodes, fitting impedance parameters with the Randles model, and establishing a quantitative relationship between bacterial concentration and inhibitor dosage using the XGBoost algorithm, the prediction error can be reduced from 4.95% to 0.46% within the inhibitor concentration range of 0.175–0.375 μL/mL after extending the bacterial incubation time from 1 to 3 h, offering a high-precision solution for microbial inhibitor analysis (Table 1).

Furthermore, a highly integrated intelligent electrochemical platform enables species classification by multi-channel monitoring of bacterial oxygen consumption kinetics and their variations under antibiotic perturbation (Figure 7C) [137]. Using dissolved oxygen sensor arrays to real-time detect the respiratory activity of five bacterial species (e.g., *E. coli*, *S. epidermidis*), combined with microscale antibiotic interference with their growth metabolism, concentration-dependent species-specific response fingerprints are generated. The resulting electrochemical signals, integrated with PCA, enable preliminary classification based on basal oxygen consumption rates, while differential inhibition of respiration by antibiotics further enhances species discrimination. This intelligent electrochemical sensing system provides a customizable high-throughput platform for rapid bacterial identification.

In virus monitoring, the binding events of biological recognition elements to target viruses trigger redox reactions on the electrode surface that generate measurable electrochemical signals. AI-assisted electrochemical biosensors enable precise and democratized virus detection through signal enhancement, multi-target analysis, and anti-interference calibration. For instance, a research team optimized ML models via feature engineering to enhance the detection performance of electrochemical biosensors for rabies virus (Figure 7D) [138]. A graphene-based microfluidic sensor combined with a portable potentiometer captures staircase cyclic voltammetry data, followed by theoretically driven feature extraction and recursive feature elimination algorithms to screen key parameters. The optimized SVM model achieves an F-score of 0.9830, significantly higher than the traditional decision tree model (F-score = 0.9394), with enhanced feature interpretability.

In recent years, the global risk of COVID-19 infection has posed severe threats to human health, causing long-term impacts on multiple organ systems. Junior et al. proposed an AI-driven electrochemical biosensor for rapid detection of SARS-CoV-2 virus in saliva samples [123]. By molecular docking, the designed biomimetic peptides were validated to exhibit high affinity (−250 kcal/mol) with the viral receptor-binding domain (RBD). After modifying electrodes with these peptides, cyclic voltammetry was used to detect virus-binding signals, and SVM was employed to optimize data analysis. This intelligent monitoring system demonstrates 100% detection sensitivity and 90% accuracy for viral samples with ≥1.8 × 10^4^ FFU/mL, providing an efficient non-invasive and portable solution for COVID-19 screening (Table 1).

### 3.3. Therapeutic Monitoring and Drug Development

**Figure 8:**
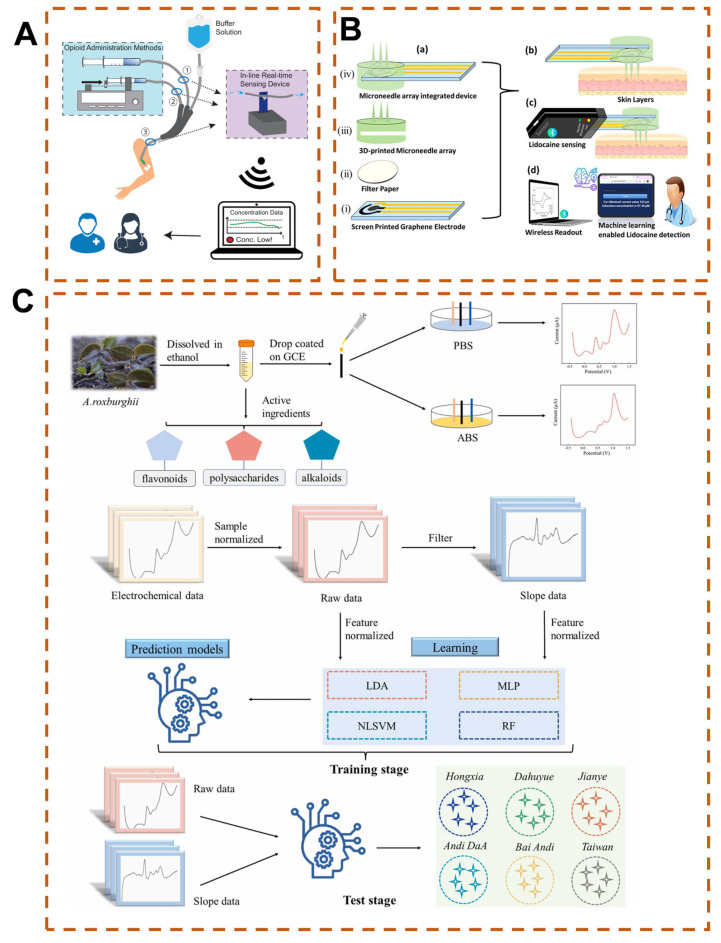
(**A**) Intelligent drug delivery system based on electrochemical sensors and ML algorithms [142], (a) Expanded view of components and stepwise assembly process of the microneedle array integrated device, (b) Fully assembled device on skin layer. (c) Fully assembled device connected with wireless sensor, (d) Wireless readout device for further analysis through machine learning enabled linear regression models followed by digital visualization of lidocaine concentration, © Copyright 2025 IEEE. (**B**) On-site electrochemical detection of lidocaine enabled by ML [144], Copyright © 2023 Elsevier Ltd. (**C**) Medicinal plant identification via electrochemical fingerprinting combined with ML algorithms [145], Copyright © 2022 Elsevier B.V.

Drug abuse has emerged as an increasingly severe social issue, not only imposing mental or physical harm on individuals but also inflicting societal damages [139,140]. Anesthetic drugs exert adverse effects on human systems, even leading to death, thereby gravely endangering human health and safety. Compared to conventional drug detection methods (e.g., chromatography, spectroscopy, and immunoassays), electrochemical techniques have garnered significant attention in pharmaceutical analysis due to their unique advantages (including real-time operation, operational simplicity, and miniaturization) [141]. For example, a portable electrochemical sensing system was designed for real-time monitoring of opioids (such as fentanyl, morphine) in intravenous infusions to prevent drug tampering risks (Figure 8A) [142]. This system integrates a miniature potentiometer with a flow cell, enabling dynamic drug detection in a simulated intravenous injection environment. After optimization by ML algorithms, the blind test classification accuracy exceeds 95%, providing a reliable tool for clinically safe medication, with an LOD of 1.26 µg/mL for fentanyl and 2.75 µg/mL for morphine.

Based on differentiated response interfaces by multi-carbon modified printed electrode arrays, Aguayo et al. proposed a modified electronic tongue sensing system for simultaneous quantitative analysis of opioid mixtures (heroin, morphine, codeine) in complex matrices (containing interferents such as caffeine and acetaminophen) [143]. The carbon electrode array modified with graphite, cobalt phthalocyanine, and palladium captures drug-specific electrochemical signals and can achieve micromolar-level detection (LOD 1.8–5.3 μM) by square wave voltammetry. The quantitative model based on partial least squares regression effectively resolves overlapping signals, with a normalized root mean square error of only 0.084 and a repeatability error ≤ 2% (*n* = 50).

Recently, the sensor platform based on microneedle arrays has provided enormous opportunities for real-time drug determination in clinical practice. The integration of AI can further predict drug administration concentrations and visualize data. Aiming at the toxic risk of lidocaine overdose, Kadian’s team reported an ML-assisted microneedle electrochemical sensing system for rapid bedside detection (Figure 8B) [144]. It noninvasively collects interstitial fluid through ultra-sharp microneedles on graphene-modified electrodes for sensitive detection of lidocaine (linear range 1–120 µM, LOD 0.13 µM). The ML model trained by experimental data can predict concentrations and achieve digital visual output through a web application, providing immediate guidance for clinically safe medication.

Antibiotics play an irreplaceable role in the treatment of infectious diseases. However, the abuse of antibiotics can lead to a series of adverse reactions, and more seriously, it is prone to generating drug resistance [135]. Biosensors can not only use indirect detection methods that reflect antibiotic concentrations by inhibition of bacterial proliferation, but also adopt direct measurement methods based on detecting redox signals during the biochemical reaction process in which antibiotics directly participate. In addition, the assistance of AI algorithms can achieve signal optimization and multi-component identification. A sensing system based on an iron ion-modified montmorillonite-modified electrode (Fe-Mt/GCE) realizes efficient detection of tetracycline hydrochloride through direct voltammetry [146]. The electrochemical response sensitivity of this sensor reaches 27.1 µA µM^−1^ cm^−2^. Compared with traditional electrodes, the modified electrode shows a higher response current intensity during rapid scanning. This is attributed to the larger specific surface area and more active sites of the modified electrode. As the concentration of doped Fe ions increases, the Mt layered surface with a larger surface area can accumulate more active sites. This accelerates the catalytic reaction rate. At the same time, the charge transfer speed is significantly improved, which macroscopically manifests as an increase in response current. Multivariate regression analysis using ML further optimizes the detection parameters, verifying the reliability of the prediction model and providing a new strategy for the development of low-cost antibiotic monitoring technologies.

In addition to drug efficacy evaluation, biosensors have also been mentioned for their applications in drug screening [147]. Due to their high sensitivity and specificity, biosensors can rapidly identify potential candidate drugs. Medicinal plants play an important role in the prevention and treatment of human diseases, and the diversity of their pharmacodynamic substances (such as phenolic compounds, ketones, alkaloids, and other electroactive components) provides potential for multi-target intervention in diseases [148,149]. These active components undergo specific oxidation reactions during voltammetric scanning, forming electrochemical fingerprints that can characterize species differences. The uniqueness of these fingerprints originates from the differences in the types and contents of active components among different species, thus laying the foundation for the chemotaxonomy of medicinal plants based on electrochemical signals. However, due to the genetic similarity of closely related species, their electrochemical fingerprints may have a high degree of homology. Different species often have identification confusion due to similar morphology and chemical compositions, making it difficult for traditional methods to accurately distinguish them [145]. AI technology significantly improves the analysis ability of fingerprints through high-dimensional data denoising and pattern recognition optimization. An ML-based electrochemical sensing system can capture the voltammetric response spectra of active components of medicinal plants, and it enhances fingerprint specificity by combining a double-buffer system (Figure 8C) [145]. Using six closely related species of *Anoectochilus roxburghii* as models, the SVM algorithm is used to analyze the response slope characteristics, achieving a species identification accuracy of 94.4%. This platform has both operational simplicity (no complex pretreatment required) and economy (low-cost electrodes), providing a new paradigm for the identification of medicinal plant resources and taxonomic research. The identification of medicinal plants further provides technical support for the research and development of traditional Chinese medicine.

In addition, the electronic tongue is an intelligent sensor that simulates the human taste system, used to identify and analyze chemical components in liquids. Its basic principles include sensor arrays, signal conversion, data processing, and pattern recognition [150]. The sensor array consists of multiple sensors with different responses to various chemical substances. The target substances to be detected undergo redox reactions on the electrode surface, producing changes in current or voltage. The generated original electrochemical signal features are subjected to signal conversion and eigenvalue extraction. Then, multivariate analysis is used to reduce the dimensionality of high-dimensional signals and map them into interpretable taste features. The taste fingerprint is a digital feature vector output by multivariate analysis. Its construction relies on the integration of physicochemical features (for example, current amplitude directly reflects taste intensity) and bionic perception features. Finally, pattern recognition algorithms such as ANNs and SVM are used to analyze these fingerprints [145]. As an example, by comparing with the data of known samples, the taste components of unknown samples are identified and classified, verifying the effectiveness of the electrochemical taste sensing system in predicting the bitterness of six drugs (such as amoxicillin) [151]. The system uses a metal phthalocyanine-modified electrode array to capture the electrochemical signals of drugs by cyclic voltammetry. After wavelet analysis and PCA for dimensionality reduction, the Euclidean distance is calculated with the acesulfame coordinate as the reference point to quantify the bitterness. After determining the centroid position by combining k-means clustering, the obtained bitterness ranking is highly consistent with human sensory evaluation, confirming the feasibility of this technology to replace traditional taste evaluation. Familiarity with the bitterness of drugs is helpful for drug development and alleviates the discomfort caused by bitterness to patients, especially children who refuse to take medicine due to bitterness. The quantitative evaluation of taste degree by intelligent electrochemical sensors has potential value in the process of drug formulation improvement.

### 3.4. Wearable and Implantable Health Monitoring

The rapid development of the Internet of Things (IoT) has driven the innovation of intelligent wearable sensors, which are characterized by flexible conformability, operational simplicity, and portability [152]. Flexible electronics technology has revolutionized the form of traditional rigid devices, enabling flexible information acquisition, transmission, and energy supply, thus significantly advancing the paradigm shift in information and communication technologies. Against this backdrop, the deep integration of wearable devices and biosensing elements has spurred the rapid development of novel biosensors and POCT diagnostic devices, providing a technological foundation for real-time disease monitoring [153]. Wearable electrochemical biosensors achieve disease diagnosis through the synergistic effect of multiple technologies, with their typical structure comprising three functional modules: a supporting substrate (often using flexible materials such as hydrogels and elastic polymers), a sensing element (capturing biomarker signals based on electrochemical principles), and a signal output unit (integrating a wireless transmission module to enable real-time cloud interaction of data) [154]. To enhance detection sensitivity and anti-interference capability, AI algorithms have been deeply integrated into the sensing system.

**Figure 9:**
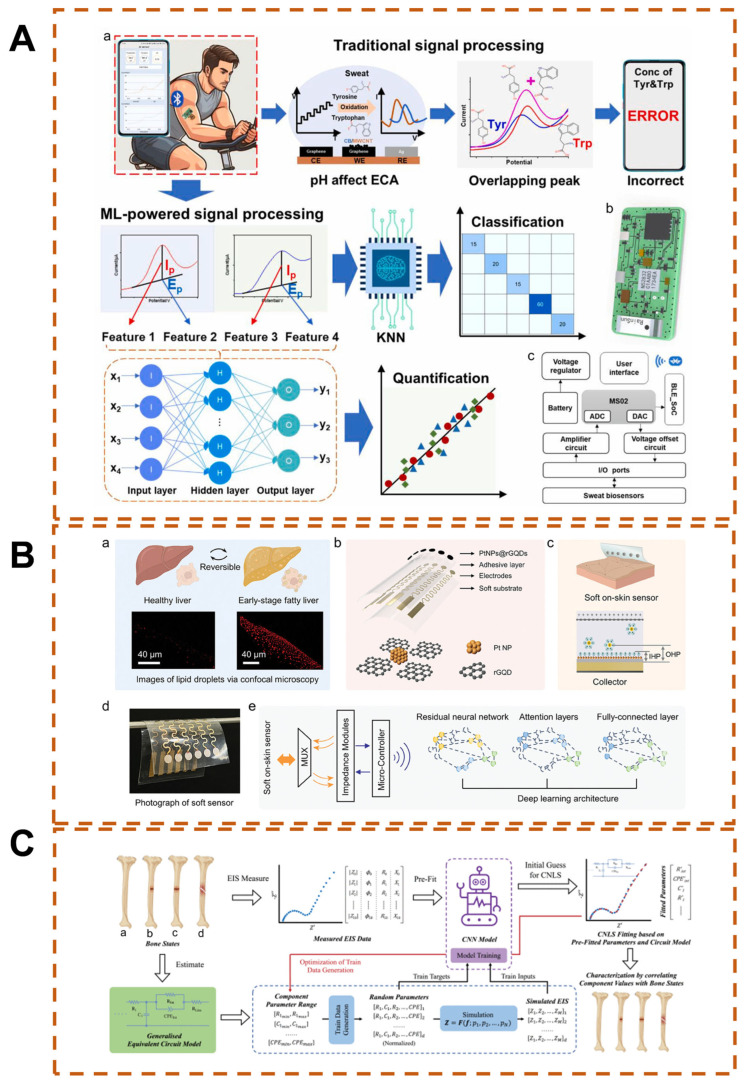
(**A**) ML-driven wearable sensing system enables monitoring of sweat biomarkers during physical activity [157], (a) Schematics of machine learning-powered signal processing overcome the limitation of traditional signal processing, achieving accurate classification and quantification. (b) Schematic of micro-electrochemical system. (c) System-level block diagram showing the signal transduction, processing, and wireless transmission from the sensors to the user interface, Copyright © 2024 Elsevier B.V; (**B**) Non-invasive detection of early-stage fatty liver via skin impedance sensors and DL [160], (a) Confocal microscopy analysis of hepatic steatosis, (b) Schematic architecture of epidermal sensor, (c) Electrochemical interface mechanism of sensing electrodes, (d) Physical prototype of soft epidermal sensor, (e) Attention-based DL for multimodal data classification, Copyright © 2024 John Wiley & Sons, Inc. (**C**) ML-assisted implantable electrochemical device for monitoring bone healing following lower limb osteosynthesis [161], (a–d) intact bone, without medial part, without medial and lateral part, and complete fractured bone, Copyright © 2024, IEEE.

Sweat contains not only abundant physiological biomarkers but also offers the advantages of easy sample collection and continuous detection, providing great convenience for real-time monitoring of various human physiological indicators [155]. For applications in sweat, non-enzymatic sensors have emerged as effective alternatives to enzymatic sensors, owing to their excellent environmental tolerance (such as insensitivity to temperature/humidity), long-term storage stability, and rapid response capability [156]. However, their practical applications are still constrained by insufficient selectivity, primarily due to the susceptibility of catalytic activity to pH fluctuations and the complexity of biological matrices. To address these limitations, a research team has developed an ML-driven wearable non-enzymatic electrochemical sensing platform (Figure 9A) [157]. This platform employs laser-induced graphene electrodes (LIGEs) to construct a flexible sensing interface and utilizes a microfluidic system for in-situ sweat collection and pH regulation. The current and potential signals are respectively correlated with the concentrations of tyrosine and tryptophan, as well as the sweat pH. The hardware layer is integrated with a miniature signal conversion module and a wireless communication module. Based on ML algorithms, it enables the analysis of tyrosine/tryptophan concentrations, transmits data to mobile terminals via Bluetooth, and displays metabolic information in real time. The microfluidic system counteracts the impact of environmental fluctuations on catalytic activity through in situ pH monitoring and correction, enhancing reliability in sports scenarios.

In addition to microfluidic systems for collecting sweat on the skin surface, hydrophilic materials can also be used to capture sweat and transport it to the sensor surface [158]. Among these, flexible porous nanomaterials excel in delivering the sweat absorbed on the skin’s surface while filtering out surface impurity particles [159]. Shahub et al. constructed a passive electrochemical-sensing ML collaborative platform by integrating double gold electrodes with a nanoporous polyamide flexible substrate, enabling dynamic trend classification of sweat cortisol concentration [124]. Based on EIS, the concentration changes in cortisol (8–140 ng/mL) are captured in the 100–500 Hz frequency band, exhibiting a significant concentration-dependent response. In conjunction with an intelligent trend recognition algorithm, a weighted KNN model is developed, which uses the response rate to changes in corticosterone concentration as a characteristic quantity to achieve classification of concentration rise and fall trends, with a verified recognition accuracy of 100% (Table 1). Flexible intelligent wearable sensing devices based on nanomaterials not only ensure the reliability and accuracy of real-time detection but also feature flexible materials with high breathability and conformal skin adhesion, significantly enhancing wearing comfort.

Wearable devices can not only monitor in real time the biomarker information (body fluid components, concentration levels, etc.) in skin surface sweat to reflect human health conditions, but can also non-invasively monitor disease status by directly collecting surface impedance spectra. The skin impedance is essentially a composite response of electrode–skin contact impedance and body tissue impedance [162]. In the low-frequency domain, the lipid bilayer hinders ion migration, presenting high capacitive reactance, and the current mainly flows through the extracellular fluid, where the impedance value reflects the state of extracellular water. In the high-frequency domain, the capacitance effect of the cell membrane weakens, the current penetrates the cell membrane into the intracellular fluid, and the impedance characterizes the intracellular hydration degree and ion concentration [163]. Therefore, fat-free tissues with high water/ion concentration and protein electrolytes form low-resistance pathways, while lipid accumulation in fat-infiltrated tissues replaces conductive body fluids, reducing ion mobility, manifesting as a significant attenuation of electrical conductivity, and the destruction of the cell membrane structure leads to a nonlinear increase in capacitive reactance [164]. Utilizing the characteristics of multi-frequency impedance spectra to identify the changes in resistance and reactance caused by liver fat infiltration provides the possibility for non-invasive detection of fatty liver. Wang et al. constructed a flexible sensor composed of platinum nanoparticles and reduced graphene quantum dots, which significantly reduces the electrode–skin contact impedance by enhancing the interfacial double-layer capacitance effect (Figure 9B) [160]. Aiming at the weak impedance characteristics of early non-alcoholic fatty liver, a multi-head self-attention DL model was developed to achieve high-specificity signal recognition, realizing an accuracy of over 97.5% in the detection of fatty liver mouse models.

Implantable sensors for circulating biomarkers in blood and interstitial fluid may provide local indicators of tissue health and organ function. This technology holds promise to revolutionize personalized medicine by facilitating early disease diagnosis, monitoring disease progression, and tailoring individual treatment regimens. Extensive existing research has reported on the real-time monitoring of neurotransmitters using implantable electrochemical sensing systems, providing highly sensitive and selective solutions for the development of brain science and the exploration of brain diseases [165,166,167]. However, implantable devices need to ensure accurate detection of biomarkers across a wide concentration range and maintain sensor stability in dynamic environments [168]. The assistance of AI algorithms can significantly enhance the signal processing performance of implantable devices. As an example, monitoring bone healing after lower limb orthopedic fixation surgery is of great significance for subsequent medical decision making. Traditional imaging methods not only increase the economic burden on patients but also lead to the risk of repeated exposure to radiation. Recent studies have leveraged implantable devices and EIS technology to develop a remote radiation-free monitoring scheme for fracture patients. Aiming at the ambiguity problem of traditional EIS in characterizing physiological/pathological features, an ML-assisted EIS analysis method for intramedullary nail fixation cases is proposed: based on equivalent circuit model theory, general distributed elements are used to construct the electrical property model of the tissue to be measured; furthermore, a CNN is used for pre-fitting the measured EIS data to achieve dominant element identification, initial guess optimization, and physiological correlation analysis (Figure 9C) [161]. The effectiveness of the method was verified through in vivo EIS measurement data of rabbit tibia after surgery. Through the high-precision matching of the measurement spectrum and the fitting curve, the key elements of the equivalent circuit model can be stably identified, and their association characteristics with the local bone state can be revealed. This method shows good potential in EIS quantitative analysis and provides a new direction for follow-up research and applications.

Overall, electrochemical biosensors assisted by AI algorithms are reshaping the boundaries of wearable and implantable health monitoring technologies through multimodal fusion and intelligent algorithm optimization. In the field of wearable devices, the combination of flexible substrates (such as hydrogels and elastic polymers) and nanomaterials (graphene, conductive polymers) constructs a highly sensitive sensing interface. By integrating ML algorithms to process EIS or current–potential signals, real-time monitoring of sweat metabolites is realized, and the anti-environmental interference ability is significantly improved, ensuring reliability in sports scenarios. In implantable devices, AI algorithms can achieve non-invasive monitoring of rehabilitation treatment and disease status by analyzing electrochemical data.

## 4. Summary and Future Perspectives

The integration of AI and electrochemical sensors has brought breakthrough innovations to the biomedical field, including the design and development of sensing elements, real-time metabolic monitoring, early disease diagnosis, and drug screening. However, the large-scale application of this technology still faces multiple challenges that require interdisciplinary collaboration to overcome. The following summarizes the future challenges and development directions from three aspects: technical bottlenecks, system integration, and application transformation.

At the data and algorithmic level, electrochemical signals are highly susceptible to noise interference, including temperature fluctuations, pH variations, and complex biological matrices. This susceptibility leads to data characterized by a low SNR and strong heterogeneity. The current absence of a unified data annotation standard significantly hampers the training efficiency of AI models. In biomarker detection, the low SNR of dynamic signals necessitates the support of higher-dimensional feature extraction algorithms. Although existing AI models demonstrate excellent performance on specific datasets, they suffer from insufficient cross-scenario generalization capabilities. For instance, when ML models developed for drug detection are applied in clinical body fluid, the accuracy decreases significantly due to the more complex interactions of biological molecules. Moreover, the “black-box” nature of DL models undermines their credibility in clinical diagnosis, necessitating the development of explainable AI. A framework is required to clarify the association between feature weights and biological mechanisms. Additionally, there is a contradiction between computing resources and real-time performance. High-precision AI models, such as DNN, demand substantial computational power, while implantable or wearable sensors require low-power edge computing. Existing systems struggle to balance these two requirements, resulting in delays in real-time monitoring.

At the hardware and component integration level, in vivo applications face critical challenges: electrodes are susceptible to protein adsorption, immune responses, or electrolyte corrosion, leading to signal drift—for example, glucose sensors requiring daily calibration. While nanomaterial modification enhances sensitivity, it often compromises long-term stability; moreover, certain materials (e.g., metal nanoparticles) pose biotoxicity risks. Miniaturization is indispensable for implantable devices, yet reducing electrode size exacerbates the SNR degradation. Simultaneously, signal crosstalk becomes prominent during multiplexed target detection. To address these, the development of novel composite materials and array-based sensor designs, coupled with feature decoupling algorithms for optimized multi-channel data analysis, is imperative. This integrated approach aims to mitigate hardware-induced limitations while enhancing analytical performance in complex biological milieus.

In the realm of clinical application and large-scale manufacturing, AI-assisted sensor systems necessitate rigorous validation through extensive clinical trials to ensure their robustness. However, inter-center data discrepancies frequently give rise to model failure, as metabolic baseline fluctuations among different populations significantly impact the interpretation of disease biomarkers. The existing regulatory framework for medical devices has not yet been fully optimized to accommodate dynamically learning AI systems, underscoring the urgent need to establish cross-regional standardized databases. High-end sensors are heavily reliant on micro-nano processing technologies, leading to exorbitant manufacturing costs. Moreover, the continuous iteration of AI models requires cloud-based support, presenting substantial deployment challenges in remote areas. These bottlenecks highlight the pressing demand for interdisciplinary solutions that integrate clinical validation protocols, regulatory innovation, and cost-effective manufacturing strategies.

Consequently, future developments in intelligent biosensors should focus on the following:

(1) Developing self-calibrating electrode materials and biomimetic interfaces, coupled with federated learning to optimize cross-scenario data utilization;
(2) Advancing lightweight NNs and edge computing chips to enable localized real-time analysis in implantable devices;
(3) Integrating electrochemical signals with genomic/proteomic data to construct multimodal AI diagnostic models;
(4) Combining microfluidic technologies with flexible electronics to develop “detection-feedback-therapy” closed-loop systems and intelligent drug delivery systems.

Furthermore, it is essential to promote collaboration among materials science, clinical medicine, and AI fields, and establish open-source databases (such as an “electrochemical sensing biomarker library”) and testing benchmarks to accelerate technological translation. These integrated approaches will address current challenges and drive the next-generation intelligent biosensors toward more reliable, efficient, and clinically applicable solutions.

## Figures and Tables

**Table 1:** Applications of AI algorithm-assisted electrochemical biosensors in biomedicine.

Algorithm Type	Electrode Materials	Test Sample	Detection Target	Sensor Optimization Results	Reference
RF	Dithiobis(succinimidyl propionate)-modified gold microelectrode	urine	IL-6, IL-8	Classification accuracy of 98.437% for disease status	[118]
Decision tree	Enzyme-based screen-printed electrode	serum	Glucose concentration	Decision criteria of sensor calibration parameters > 0.9; coefficient of determination for glucose concentration = 0.828	[120]
RBF-ANN	Multi-walled carbon nanotube-ionic liquid co-modified graphite electrode	blood	Red blood cell count	Performance comparable to automated blood cell counters	[121]
XGBoost	Multi-walled carbon nanotube–Prussian blue–gold nanoparticle composite material	*Escherichia coli*	Antiseptic antimicrobial efficacy	Prediction error range of bacteriostatic concentration under varying inhibitor concentrations (0.46–4.95%)	[122]
SVM	Biomimetic peptide-modified screen-printed electrode	saliva	SARS-CoV-2	Achieved 100% sensitivity, 80% specificity, and 90% accuracy in viral detection of infected saliva	[123]
KNN	Nanoporous polyamide flexible substrate integrated with dual gold electrodes	sweat	Corticosterone concentration	100% accuracy achieved in detecting corticosterone concentration changes	[124]
ANNs	Electrospun barium titanate nanofiber film	skin-mounted wearable devices	Diagnosis of sarcopenia	Diagnostic accuracy of sarcopenia detection reaches 92.9% and 98.1% in male and female populations, respectively	[125]

## Data Availability

Data are contained within the article.

## Abbreviations

The following abbreviations are used in this manuscript:

AI	artificial intelligence
COVID-19	coronavirus disease 2019
SERS	surface-enhanced Raman spectroscopy
FDA	Food and Drug Administration
ML	machine learning
SVM	support vector machine
PPE	poly(p-aryl acetylene)
AIE	aggregation-induced emission
RF	random forest
PCA	principal component analysis
HCA	hierarchical cluster analysis
LDA	linear discriminant analysis
DL	deep learning
ANNs	artificial neural networks
DNN	deep neural network
NNs	neural networks
MLP	multi-layer perceptron
CNN	convolutional neural network
RNN	recurrent neural network
MIPs	molecularly imprinted polymers
KNN	k-nearest neighbors
SNR	signal-to-noise ratio
POCT	point-of-care testing
BPNN	backpropagation neural network
RBF-SVC	radial basis function support vector classifier
SISSO	Sure Independence Screening and Sparsifying Operator
EIS	electrochemical impedance spectroscopy
IVD	in vitro diagnostics
RBF	radial basis function
VOCs	volatile organic compounds
LOD	limit of detection
RBF-PLS	radial basis function–partial least squares
LS-SVM	least squares–support vector machine
RBF-ANN	radial basis function–artificial neural network
AMR	antimicrobial resistance
RBD	receptor-binding domain
IoT	Internet of Things
LIGE	laser-induced graphene electrode
PVA	polyvinyl alcohol
